# Two-Stage Gene Therapy (VEGF, HGF and ANG1 Plasmids) as Adjunctive Therapy in the Treatment of Critical Lower Limb Ischemia in Diabetic Foot Syndrome

**DOI:** 10.3390/ijerph191912818

**Published:** 2022-10-06

**Authors:** Piotr Barć, Maciej Antkiewicz, Katarzyna Frączkowska-Sioma, Diana Kupczyńska, Paweł Lubieniecki, Wojciech Witkiewicz, Małgorzata Małodobra-Mazur, Dagmara Baczyńska, Dariusz Janczak, Jan Paweł Skóra

**Affiliations:** 1 Department and Clinic of Vascular, General and Transplantation Surgery, Wroclaw Medical University, Borowska 213, 50-556 Wroclaw, Poland; 2 Department of Angiology, Hypertension and Diabetology, Wroclaw Medical University, Borowska 213, 50-556 Wroclaw, Poland; 3 Research and Development Center, Regional Specialized Hospital in Wroclaw, Kamienskiego 73a, 51-124 Wroclaw, Poland; 4 Department of Forensic Medicine, Division of Molecular Techniques, Wroclaw Medical University, M. Curie-Sklodowskiej 52, 50-369 Wroclaw, Poland; 5 Department of Molecular and Cellular Biology, Wroclaw Medical University, Borowska 211A, 50-556 Wrocław, Poland

**Keywords:** gene therapy, ANG1, VEGF, HGF

## Abstract

One of the most serious problems in people with diabetes is diabetic foot syndrome. Due to the peripheral location of atherosclerotic lesions in the arterial system of the lower extremities, endovascular treatment plays a dominant role. However, carrying out these procedures is not always possible and does not always bring the expected results. Gene therapy, which stimulates angiogenesis, improves not only the inflow from the proximal limb but also the blood redistribution in individual angiosomes. Due to the encouraging results of sequential treatment consisting of intramuscular injections of VEGF/HGF bicistronic plasmids followed by a month of ANG1 plasmids, we decided to use the described method for the treatment of critical ischemia of the lower limbs in the course of diabetes and, more specifically, in diabetic foot syndrome. Twenty-four patients meeting the inclusion criteria were enrolled in the study. They were randomly divided into two equal groups. The first group of patients was subjected to gene therapy, where the patients received intramuscular injections of pIRES/VEGF165/HGF plasmids and 1 month of ANG-1 plasmids. The remaining patients constituted the control group. Gene therapy was well tolerated by most patients. The wounds healed significantly better in Group 1. The minimal value of ABI increased significantly in Group 1 from 0.44 ± 0.14 (± standard deviation) to 0.47 ± 0.12 (with *p* = 0.028) at the end of the study. There were no significant differences in the control group. In the gene treatment group, PtcO2 increased significantly (from 28.71 ± 10.89 mmHg to 33.9 ± 6.33 mmHg with *p* = 0.001), while in Group 2, no statistically significant changes were found. The observed resting pain decreased significantly in both groups (Group 1 decreased from 6.80 ± 1.48 to 2.10 ± 1.10; *p* < 0.001; the control group decreased from 7.44 ± 1.42 to 3.78 ± 1.64 with *p* < 0.001). In our study, we evaluated the effectiveness of gene therapy with the growth factors described above in patients with CLI in the course of complicated DM. The therapy was shown to be effective with minimal side effects. No serious complications were observed.

## 1. Introduction

Diabetes mellitus, a disease considered by many to be a global epidemic of the 21st century, is the first noninfectious disease that has spread all over the world, and it poses a number of challenges for physicians. The International Diabetes Federation (IDF) predicts that the number of diabetics in the world may increase worldwide from 536.6 million people to 783.2 million in 2045 [1]. The main reason for this is the sharp increase in the incidence in developing countries [2].

Poorly controlled Type 2 diabetes causes a number of complications related to damage to the large coronary or cerebral vessels, leading to heart attacks or strokes. The involvement of the smaller vessels, which is typical of the degenerative nature of metabolically decompensated diabetes, leads to diabetic retinopathy, nephropathy or neuropathy. The latter largely contributes to the development of diabetic foot syndrome, the most common nontraumatic cause of limb amputation.

One of the most serious problems in people with diabetes is diabetic foot syndrome. According to the definition of the International Working Group on the Diabetic Foot (IWGDF), it involves an infection, deep tissue ulceration and/or destruction that is associated with neurological disorders and peripheral vascular disease to various degrees of advancement in the lower extremities (below the ankle) in people with diabetes. The risk of developing such ulcers throughout life of a diabetic patient is up to 25% [3] and ulcers can become infected over time. Infections are the most common reason for hospitalization among this group of patients [4]. Additionally, the probability of amputation is 30–40 times higher compared with diabetes-free groups of patients [5,6].

As a result of the dominant neuropathic component, the patient does not experience cuts, injuries to the feet or badly selected (too tight) footwear for a long time. The final stage of diabetic foot disease is critical lower limb ischemia, which develops over time due to the progression of microangiopathic and macroangiopathic complications.

Macroangiopathic lesions qualify for revascularization treatment. Vascular procedures, mainly endovascular, give a chance to improve tissue perfusion and healing and to avoid limb amputation. Due to the peripheral location of atherosclerotic lesions in the arterial system of the lower extremities, endovascular treatment plays a dominant role. However, carrying out these procedures is not always possible and does not always bring the expected results because of the lack of technical possibilities and also the fact that improving the inflow to better supplied angiosomes will not necessarily allow sufficient perfusion of those where necrotic changes or ulcerations have occurred [7,8]. Due to the lack of dedicated treatment for “patients not eligible for revascularization” (NCR), metabolic control of diabetes, statin intake, antiplatelet drugs, smoking cessation and local treatment are the only therapeutic options. Despite the use of conservative therapy, the annual risk of amputation in this group of patients is 40%, and the mortality rate is almost 20% [7,9]. Therefore, there is a need to search for new solutions that would improve the outcomes of diabetic foot syndrome treatments. Apart from the continuous development of the existing measures (improvements in the surgical techniques, the introduction of new devices allowing practitioners to extend the scope of invasive treatment and the introduction of new drugs), an attempt to induce angiogenesis seems to be an interesting alternative. For about 20 years, attempts have been made to treat critical limb ischemia with gene therapy by using plasmids encoding growth factors or autogenous stem cells obtained from bone marrow or adipose tissue [10,11].

Gene therapy, which stimulates angiogenesis, improves not only the inflow from the proximal limb but also the blood redistribution in individual angiosomes. Since 2004, we have been conducting research on gene therapy for critical ischemia of the lower limbs at the Department and Clinic of Vascular Surgery at the Medical University of Wrocław [12,13].

After experimental studies on animals (several series) and testing the angiopoietic potential of growth factor plasmids (including VEGF, HGF and ANG), promising treatments were started in patients suffering from critical ischemia of the lower limbs in the course of atherosclerosis, then Burger disease. Initially, therapy with the VEGF plasmid was performed, then sequential treatment consisting of the intramuscular administration of VEGF/HGF bicistronic plasmids, then a month of ANG1 plasmids. Due to the encouraging results, including a significant reduction in the amputation rate (by approximately 50%), we decided to apply the method for the treatment of lower limb ischemia in the course of diabetes and, more specifically, diabetic foot syndrome.

## 2. Materials and Methods

### 2.1. Preparation of Plasmid DNA

**Figure 1:**
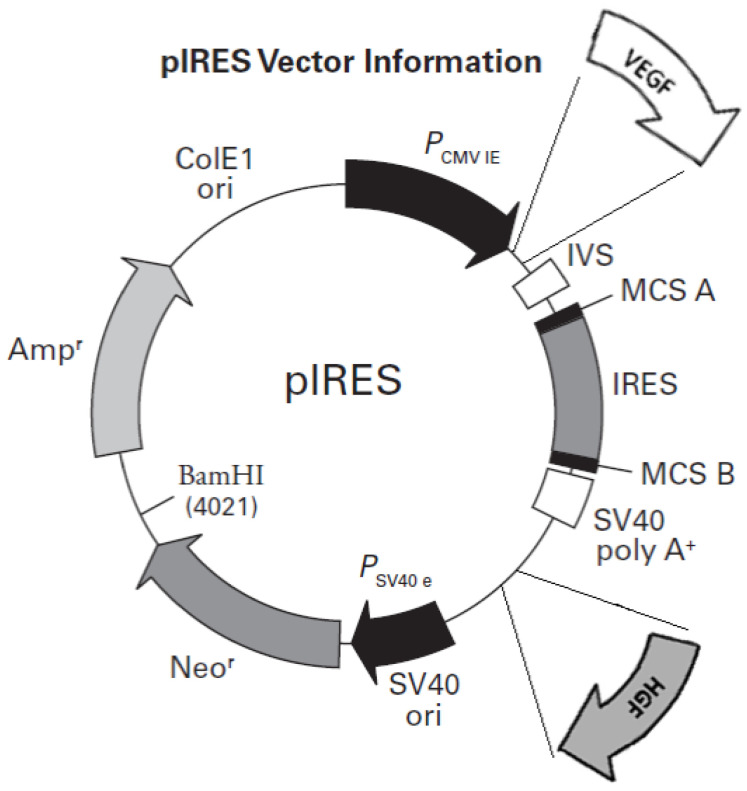
Schematic representation of the plasmids used with the two cloned transcription factor genes used for gene therapy (VEGF and HGF).

The bicistronic plasmid was designed on the basis of our previous research. The CMV promoter was used because of its significant transfection efficiency in vitro [14]. A schematic diagram of the structure of the plasmid is presented in Figure 1. Human cDNA for HFG and VEGF165 were prepared as previously described [15]. Both cDNAs were cloned into the bicistronic plasmid pIRES using restriction enzymes. All the pIRES/VEGF165/HGF plasmids thus obtained were purified and dissolved and their pyrogenicity was excluded. The ANG-1 plasmid was prepared analogously. The whole process has already been described in detail [16].

### 2.2. Patient Cohort

Inclusion criteria for this study were (1) Type 2 DM; (2) critical limb ischemia (III–IV grade according to Fontaine’s classification), i.e., pain at rest and necrosis, nonhealing ischemic ulcers or gangrene persisting for at least 12 weeks; (3) no effect of conventional critical limb ischemia treatment for at least 4 weeks; and (4) the inability to perform revascularization. Patients with the following conditions were excluded: severe retinopathy, AMD (age-related macular degeneration), end-stage renal disease, angina, NYHA Grade III or IV heart failure, liver dysfunction (Child–Pugh B or C), a history of active malignant neoplastic disease or neoplastic disease during oncological follow-up (up to 5 years), or the inability to stand or walk without assistance.

Patients eligible for urgent amputation procedures, those with very extensive necrotic lesions or ulcerations, patients with no prognosis for mobilization, patients in a severe condition, and those who were unable to give informed consent for the study were also excluded.

Twenty-four patients meeting the inclusion criteria, who gave informed consent to the experimental treatment, were enrolled in the study. They were randomly divided into two equal groups. The first group consisted of 12 patients (seven men and five women aged 55 to 74 years, with a mean age of 64.4 years) who were subjected to gene therapy. The remaining patients (seven men and five women aged 53 to 75 years, with a mean age 65.2) did not receive plasmids as the control group (Group 2). The mean level of hemoglobin A1c was 7.9% (range 6.2–9.4%).

The follow-up time was 12 weeks. All of the patients received conventional diabetic foot disease therapy (antiplatelet and statin drugs, glucose level maintenance, exercise training, wound debridement and antibiotic therapy) if they showed clinical signs of tissue infection.

### 2.3. Administration of Plasmids

The first step involved intramuscular injections of pIRES/VEGF165/HGF. Injections were performed in the ischemic lower limb below and above the knee level. Each patient in the first group received 4 mg of the bicistronic plasmid (approximately 20 mL). The intramuscular injection sites were based on our previous research and data from the literature [17]. The volume of each injection was approximately 0.25 mL (roughly 80 injections, 2–4 cm deep, into the ischemic limb muscles along each of the three major arterial trunks of the lower leg, and into the borderline zone between angiosomes). The administration time did not exceed 20 min. One month later, the ANG-1 plasmid was administered in an analogous manner. Patients from the control group were given saline instead of the plasmid in the same manner.

### 2.4. Clinical Evaluation

The observation period was 12 weeks. Three months after the onset of treatment, all patients were tested for hemoglobin levels, including hemoglobin A1c, thrombocytes, leukocytes, C-reactive protein and creatinine. In addition, vital signs, weight, body temperature, blood pressure and heart rate were measured. Necrotic changes (their extent, demarcation and infiltration of adjacent tissues) and ulcerations (surfaces, depth and activity – granulation or anergic) were assessed and assigned according to a numerical scale as follows: 0, without ulceration or necrosis; 1, A shallow ulcer without necrosis; 2, bone/ligament at the bottom of the ulcer, or necrosis limited to the toes; 3, extensive ulceration/necrosis of the metatarsus or tarsus. All changes WERE documented with photos and descriptions. Moreover, the general condition of the patients was assessed.

### 2.5. Ankle–Brachial Index (ABI)

ABIs were recorded 1 week before, AND 1 and 3 months after the start of treatment in both groups of patients. It was calculated as the ratio of the highest pressure from the anterior or posterior tibial artery to the highest systolic pressure on the upper arm.

### 2.6. Resting Pain

Resting pain was assessed during qualification for treatment, before plasmid administration and 3 months after the initiation of treatment in both groups of patients. Pain intensity was assessed using a visual analog scale (VAS, 0–10).

### 2.7. Transcutaneous Oxygen Pressure (TcPO2) Measurements

TcPO2 was tested during qualification for the procedure, before plasmid administration and 3 months after the onset of gene therapy in both groups. The Medicap Precise 8001 device was used for these measurements. The test turned out to be the most sensitive of the additional tests used, and its results were highly correlated with the clinical effects of the treatment. Its disadvantage and the only significant limitation was the long duration of the measurement.

### 2.8. Statistical Analysis

Statistica 13.3 (StatSoft, Krakow, Poland) was used for statistical analysis. The work presents the results classified as the so-called industry statistics, using both descriptive statistics (age, gender, yes/no data) and mathematical statistics.

Based on data from the literature, it was assumed that the sample error was about 2% and the confidence level was about 96%. The Shapiro–Wilk test for normality was performed, followed by Student’s *t-*test for comparisons between the obtained coefficients. Wilcoxon’s test was used for nonparametric analyses. The significance level was set at a *p*-value of less than 0.05.

## 3. Results

### 3.1. Clinical Follow-Up

Gene therapy was well tolerated in most patients. No significant side effects were observed. In three patients, slight swelling occurred (for up to 2 days); in one of them, skin reddening was noted, and the patient was withdrawn after one day. During the studies, none of the patients in both groups had significant changes in their vital signs or laboratory parameters.

**Figure 2:**
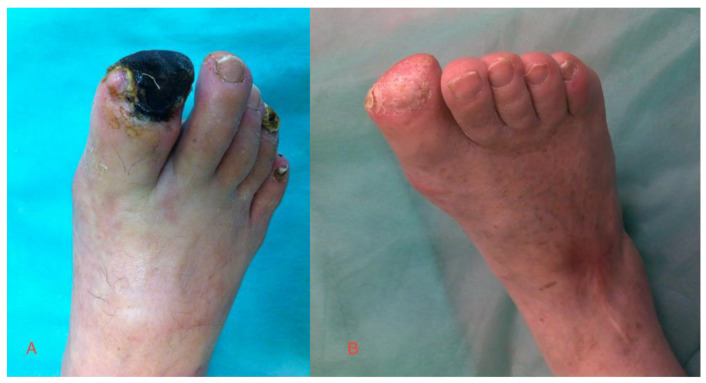
Toe necrosis in a Group 1 patient (**A**) before treatment and (**B**) after 3 months.

**Figure 3:**
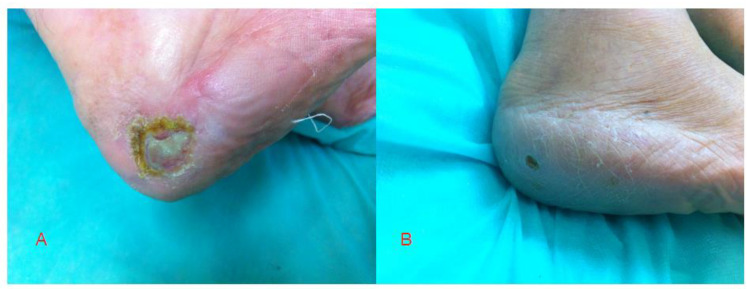
Ulcer healing in a Group 1 patient (**A**) before treatment and (**B**) after 3 months.

Surgical debridement of the necrotic tissue was performed in some patients. Below-knee amputation was finally performed in two patients in Group 1 (gene therapy) and in three patients in Group 2. The wounds healed significantly better (except for the two patients who underwent amputations) in Group 1. A statistical analysis of the healing process is presented in Table 1. The photos show the effects of the applied gene treatment (Figure 2 and Figure 3).

### 3.2. ABI Results

On average, ABI increased significantly in Group 1 from 0.44 ± 0.14 to 0.47 ± 0.12 at the end of the study (Table 1). In Group 2, the ABI did not differ significantly and there was no significant increase after 3 months of the treatment. Five patients (two in Group 1 and three in Group 2) had no follow-up ABI study because of amputation.

### 3.3. PtcO2 Results

PtcO2 is an objective and precise parameter for the assessment of tissue perfusion. In the gene treatment group, PtcO2 increased significantly from 28.71 ± 10.89 mmHg to 33.9 ± 6.33 mmHg, while in Group 2, no statistical significance was seen (Table 1).

### 3.4. Resting Pain (VAS)

The observed resting pain decreased significantly in both groups. The scores of patients in Group 1 decreased from 6.80 ± 1.48 to 2.10 ± 1.10, and those of patients in Group 2 decreased from 7.44 ± 1.42 to 3.78 ± 1.64 (Table 1).

## 4. Discussion

Each year, PAD leads to the development of 1000 new cases of CLI per million people, affecting men three times more often than women [7,18,19]. In Poland, 90,000 people suffer from complicated diabetes PAD and are at risk of amputation. This procedure is performed in 15.5 thousand people per year, of which one-third are the so-called large amputations (above the ankle). Poland is, infamously, in the tenth place out of 31 European countries in terms of the number of limb amputations in adult diabetics. Between 2014 and 2018, the number of amputations due to diabetes increased by more than one-fifth and the cost of amputations increased by 44% [20]. All this makes PAD and, consequently, CLI a serious threat for every diabetic, often leading to amputation and even life-threatening conditions. Frequently, these patients are not candidates for surgery. Conservative pharmacological therapy, rehabilitation, and dietary and local treatment are always carried out, which is often insufficient for limb salvage. In view of the facts above, new therapeutic options are constantly being searched for. Gene therapy is one of them.

Angiogenesis is the process of creating new blood vessels from existing ones. As a multi-stage process, it is strictly regulated by a number of proangiogenic and antiangiogenic factors [21,22,23]. Our study was designed to test the effectiveness of the treatment of CLI in the course of DM after the injection of proangiogenic factors, thus increasing their concentration in infected tissues. The choice of specific plasmids was dictated by the results of animal experiments and data from the literature [24,25,26].

In models of new blood vessel formation, the growth factors VEGF and HGF are believed to be involved in the initial stages, especially vasculogenesis (the formation of capillaries from progenitor cells) and angiogenesis (the formation of vessels from existing capillaries) [24,25,26], while ANG-1 is involved in the later stages of maturation of newly formed vessels and arteriogenesis (the formation of mature vessels, and differentiation into arteries and veins) [27,28,29]. These reports were confirmed in our animal model studies.

VEGF, one of the key proangiogenic factors, is the main regulator of angiogenesis and vasculogenesis [30]. It is, inter alia, an inducer of the increase in vascular permeability [31], which causes the leakage of plasma proteins into the extracellular space and then organization of the extracellular matrix. Matrix formation stimulates migration and vessel formation [32]. Subsequent studies, however, have proved the low effectiveness of using single-VEGF therapy [21,26]. Therefore, in our study, we used HGF, which has so far proved to be the best in similar studies, but again not as a single therapy. The release of HGF into the extracellular matrix is increased, among others, by VEGF activity during neoangiogenesis [21]. Apart from VEGF, angiopoietin 1 and 2 (Ang-1) also play an important role in the maturation of blood vessels. Ang-1, acting through the Tie-2 receptor, remodels vessels and stabilizes mature vessels through interactions between endothelial cells and the surrounding support cells [33].

In our study, we assessed the efficacy and safety of gene therapy with the growth factors described above in patients with CLI in the course of DM. The therapy was shown to be effective with minimal side effects. The ABI in patients treated with gene therapy significantly improved, the resting pain assessed with the use of the VAS scale decreased significantly (more than in the control group) after 3 months, and there were fewer amputations and greater progress in local treatment. The safety of the therapy is worth mentioning. No serious complications were observed.

It should be mentioned that gene therapy was not an obstacle to performing other procedures, including vascular surgery. It can be a very good complement to existing methods.

## 5. Conclusions

Our study has limitations. The control group was not double-blinded. Moreover, the parameters measured during the test may not be fully objective. In the case of the ABI, the results may not be accurate due to the stiffness of the arteries and the inability to relieve the systolic pressure by inflating the air cuff. On the other hand, the VAS scale is an individual, fully subjective assessment of the symptoms by the patient [34].

Despite this, our study, in the statistical analyses, showed significant improvements in patients receiving gene therapy and improved their quality of life. It seems that pIRES/VEGF165/HGF/ANG-1 therapy with bicistronic plasmids is a safe and effective additional treatment for patients with CLI and DM. It is important to maximize the treatment of diabetes. Our study showed that in the gene therapy group, patients with well-controlled diabetes had the best results. The cost of therapy for one patient was not less than USD1000 and is a significantly lower burden on the state budget than caring for a patient with significant disability or after limb amputation

## Figures and Tables

**Table 1:** Gene therapy outcomes.

		Before Treatment (±SD)	After 3 Months (±SD)	*p*-Value
Wound classification (cm)	Group 1	1.80 ± 0.42	1.40 ± 0.52	0.037 *
	Group 2 (control)	1.78 ± 0.44	1.67 ± 0.50	0.347
ABI	Group 1	0.44 ± 0.14	0.47 ± 0.12	0.028 *
	Group 2 (control)	0.46 ± 0.18	0.49 ± 0.17	0.175
PtcO2 (mmHg)	Group 1	26.90 ± 8.99	33.9 ± 6.33	0.001 *
	Group 2 (control)	28.71 ± 10.89	32.0 ± 8.81	0.058
VAS	Group 1	6.80 ± 1.48	2.10 ± 1.10	<0.001 *
	Group 2 (control)	7.44 ± 1.42	3.78 ± 1.64	<0.001 *

Wound classification; 0, without ulceration or necrosis; 1, a shallow ulcer without necrosis; 2 bone/ligament at the bottom of the ulcer, or necrosis limited to the toes; 3 extensive ulceration/necrosis of the metatarsus or tarsus. ABI, ankle–brachial index; PtcO2, transcutaneous oxygen pressure measurement; VAS, 10-point pain scale. * Statistically significant results.

## Data Availability

The data are available upon request.
